# Comparative analysis of sport-related concussions in stick-handling sports

**DOI:** 10.1016/j.jsampl.2026.100150

**Published:** 2026-07-11

**Authors:** Clayton R. Baker, Nick De Oliveira, Justin Huang, Avi N. Albert, Kristen L. Williams, Scott L. Zuckerman, Douglas P. Terry

**Affiliations:** a Vanderbilt University School of Medicine, Nashville, TN, USA; b Vanderbilt Sports Concussion Center, Vanderbilt University Medical Center, Nashville, TN, USA; c Meharry Medical College School of Medicine, Nashville, TN, USA; d Department of Neurological Surgery, Vanderbilt University Medical Center, Nashville, TN, USA

**Keywords:** Lacrosse, Hockey, Sport-Related Concussion

## Abstract

**Introduction:**

Concussions are common in stick-handling sports, including lacrosse or hockey, where high-speed play combines player contact with equipment- and projectile-related impacts. In a cohort of lacrosse and hockey athletes who sustained a sport-related concussion (SRC), this study compared: 1) injury mechanisms, 2) initial symptom burden, and 3) recovery timelines between the sports.

**Methods:**

In this retrospective cohort study of patients (12–23 years) with SRC from lacrosse or hockey, injury mechanism was categorized as player-to-surface, player-to-player, player-to-stick, or player-to-ball/puck. Outcomes included initial Post-Concussion Symptom Scale (PCSS) score and time to return-to-learn (RTL), symptom resolution (SR), and return-to-play (RTP). Nonparametric tests compared groups, and multivariable linear regression evaluated associations between sport and injury mechanism with outcomes, adjusting for age, sex, and prior concussions.

**Results:**

Of 80 athletes (median age = 16.6), 37 played lacrosse (60% male) and 43 played hockey (86% male). Injury mechanism differed by sport (p = 0.03): lacrosse had more player-to-surface (35.1% vs. 27.9%), player-to-stick (24.3% vs. 11.6%), and player-to-ball/puck (21.6% vs 9.3%) injuries and less player-to-player (18.9% vs. 48.8%) injuries than hockey. Initial PCSS scores were higher after stick-related injuries (median = 47.5) than other mechanisms (surface = 26.0, player = 20.0, ball/puck = 31.0; p = 0.02), though mechanism was not a significant predictor in multivariable regression. Time to RTL, SR, and RTP did not differ by sport, injury mechanism, or competition setting.

**Conclusion:**

Symptom severity and recovery timelines were similar between sports and setting. Stick and ball/puck impacts were associated with greater initial symptom burden and longer RTL, which may warrant closer early monitoring and enhanced school accommodations.

## 1 Introduction

Concussions and musculoskeletal injuries are common and concerning issues in stick-handling sports such as lacrosse and ice hockey [[1], [2], [3]]. These sports are distinct in that they not only involve high-speed gameplay and frequent physical contact, but also the use of sticks and hard projectiles (puck, ball, etc.) as essential components of play [1,[4], [5], [6], [7]]. This equipment introduces additional mechanisms of injury, such as stick-to-head contact or being struck by a ball or puck, and may also reduce athletes’ situational awareness during play, which potentially increases injury risk. Prior research has identified shared injury patterns between hockey and lacrosse—often based on physical player-to-player contact or equipment (stick, ball/puck) [4,5]. Even with similar opportunities for injury, there have been notable differences based on sex, age, and competition level [5,[8], [9], [10]]. For example, younger lacrosse and hockey athletes are more likely to sustain facial and head injuries, and male athletes sustain more upper extremity injuries while female lacrosse athletes sustain more equipment-related facial injuries [5]. Recognizing these distinctions in injury patterns is essential for developing targeted and effective injury prevention strategies.

Concussions are prevalent in stick-handling sports [11,12]. Epidemiological data indicate that concussion rates in boys' lacrosse and ice hockey are among the highest of all high school sports [2,13]. Of all hockey injuries, one comparative study found that 21% were concussions [13]. Not surprisingly, concussion rates vary by age, sex, mechanism of injury, and sport [2,5,8,14]. Regarding mechanism of injury, in boys' lacrosse, concussions are primarily due to direct player-to-player contact, often involving unanticipated collisions and head-to-head impacts [15,16]. In girls' lacrosse, where contact rules are more restrictive and the athletes do not wear helmets, there is a higher proportion of both general head injuries and concussions resulting from stick or ball contact [10,16]. Similar sex-based distinctions exist in hockey: male athletes are more frequently concussed via physical contact, while female athletes experience a higher burden of equipment- and projectile-related concussions [5,17]. These patterns persist despite variations in protective gear and enforcement of contact rules, highlighting the influence of sport-specific culture and sex-specific biomechanics on injury risk [17,18]. Further, pediatric and adolescent populations may be especially vulnerable; in men's lacrosse, youth players had higher concussion rates than high school and collegiate athletes and sustained more head/face trauma overall [14].

Taken together, prior literature highlights both the common and distinct injury risks faced by athletes in stick-handling sports. Building on these sport-specific findings, broader investigations across other high-risk contact sports further emphasize the need for cross-disciplinary analysis of stick handling sports. The present study focuses on youth and adolescent lacrosse and hockey players to compare 1) injury mechanisms, 2) initial symptom burden, and 3) recovery timeline across and between both sports.

## 2 Methods

### 2.1 Study design

A retrospective cohort study was conducted using patients who presented to a regional sports concussion center in Middle Tennessee between November 2017 and April 2022 (n = 2059 athletes across all sports). The diagnosis of sport-related concussion (SRC) was based on ICD-9 and ICD-10 concussion codes (850.∗ and S06.0X∗∗, respectively). All patients initially identified with SRC were manually reviewed by trained individuals. Institutional review board (IRB) approval was obtained, and the study was exempt from consent requirements (IRB #222278).

### 2.2 Participants

The study included lacrosse and ice hockey athletes aged 12–23 years who were diagnosed with an SRC at our multidisciplinary concussion clinic. Patients younger than 12 years or older than 23 years at the time of injury, those with positive neuroimaging findings, and those with a non-sport related mechanism of injury were excluded. Data were obtained through manual chart reviews and entered into a REDCap database. Follow-up calls were conducted to collect missing data identified during the chart review.

### 2.3 Exposure variable

**Fig. 1:**
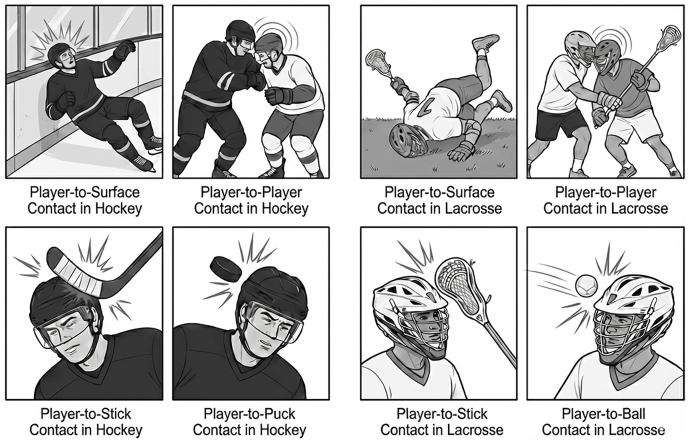
Artificial intelligence generated visual representation of injury mechanisms.

Mechanism of injury was the primary independent variable, classified based on the type of event leading to the concussion. Categories, which included player-to-surface contact (i.e. ground, ice, wall-boards), player-to-player contact, player-to-stick contact, and player-to-ball/puck contact, were established based on prior literature [10,15,18,19]. Player-to-surface contact was defined by the player's head making contact with the ground, ice, wallboards or other environmental object. Player-to-player contact involved direct head-to-head or head-to-body impacts. If a player was hit, and it was unclear whether their head was struck by the opponent or the surface, it was coded as player-to-player contact. Stick or ball impacts referred to concussions resulting directly from being struck by equipment or projectiles. Fig. 1 visualizes these injury mechanisms [20].

### 2.4 Outcome variables

The primary outcome was initial symptom severity. Symptom severity was measured at the first clinic visit via the Post-Concussion Symptom Scale (PCSS), which is a 22-item self-reported measure scored on a 7-point Likert scale. Secondary outcomes were recovery metrics, which included time to return to learn (RTL), symptom resolution (SR), and return to play (RTP). All recovery timelines were measured in days post-injury. RTL was defined as the time until the athlete resumed any amount of academic activity. SR was determined when athletes returned to their baseline symptom level or reported being asymptomatic. RTP was identified when athletes received full medical clearance to resume sport activities in an unrestricted manner. Any recovery greater than 90 days was truncated to 90 days to prevent outliers from overly influencing statistical models.

### 2.5 Statistical analysis

Descriptive statistics summarized demographic data, medical history, concussion characteristics, and recovery metrics. Continuous variables were reported as median with interquartile range (IQR) and analyzed with Mann–Whitney U-tests and Kruskal–Wallis tests. Post-hoc Dunn tests with Holm correction were performed when there was a significant Kruskal–Wallis test. Categorical variables were reported as frequencies and analyzed with Chi-squared tests. Multivariable linear regression analyses evaluated the predictive relationship between concussion mechanisms and outcome variables (initial PCSS, RTL, SR, RTP). Reference categories were established as player-to-surface contact for mechanism analyses and hockey as reference for sport analysis. Covariates included age, sex (reference: male), and number of prior concussions. A sub-analysis compared the male athletes from hockey to the male athletes from lacrosse. A descriptive sub-analysis of all female athletes was performed, though comparative statistics were not performed due to sample size. Missing or unclassifiable data (i.e., values recorded as unknown) were handled through casewise deletion across all analyses (Chi-square, Mann–Whitney U, Kruskal–Wallis, and regression models). Given the modest sample size and presence of missing data, Little's Missing Completely at Random (MCAR) test was performed, with non-significant findings suggesting data was missing in a random manner. Only complete cases for the variables under comparison were included in each respective test. Statistical significance was established at p ≤ 0.05. All analyses were conducted using Python version 3.13.

## 3 Results

### 3.1 Sample characteristics

Of 2059 patients who were treated for a concussion, there were a total of 39 lacrosse players and 44 hockey players. Of 39 lacrosse players, 2 athletes had non-sport injury mechanisms, leaving 37 lacrosse athletes in the study. Of the 44 hockey players, 1 athlete was excluded due to structural brain injury on imaging, leaving 43 hockey athletes in the study. The final total sample was 80 hockey and lacrosse athletes. This sample was predominantly male (73.8%), though hockey (86.0%) had significantly more males than lacrosse (59.5%, *p* = *0.01*).

All included athletes were between 12 and 23 years old at the time of concussion. Athletes from both lacrosse (median: 16.4 y) and hockey (median: 16.6 y) had similar ages at time of concussion (*p* = *0.34*). Both lacrosse (89.2%) and hockey (95.3%) athletes were predominantly white. While lacrosse (59.5%) and hockey (86.0%) cohorts were predominantly male, hockey had a significantly greater proportion of males (*p* = *0.01*). Lacrosse athletes (median: 1.0) had more prior concussions than hockey athletes (median: 0.0, *p* = *0.05*). Compared to hockey (4.7%), a higher proportion of lacrosse athletes (21.6%) had a preinjury psychiatric disorder (*p* = *0.05*). Other medical history characteristics, including personal history of attention-deficit/hyperactivity disorder (ADHD), and migraines as well as family history of migraine or psychological disorder, were similar between the two sports. Full sample characteristics can be found in Table 1.

**Table 1:** Sample characteristics between lacrosse and hockey athletes

	Lacrosse (*n* = 37)	Hockey (*n* = 43)	Statistics
**Demographics**			
Age at concussion (Md [IQR])	16.4 [15.3–17.7]	16.6 [14.5–17.8]	U: 696.0; *p* = 0.34
Sex: Male (*n*, %)	22, 59.5%	37, 86.0%	**χ^2^: 6.0; *p*** = **0.01**
Race: White (*n*, %)	33, 89.2%	41, 95.3%	χ^2^: 0.4; *p* = 0.54
**Medical history**			
ADHD (*n*, %)	4, 10.8%	5, 11.6%	χ^2^: 0.0; *p* = 1.00
Migraine history (*n*, %)	2, 5.4%	3, 7.0%	χ^2^: 0.0; *p* = 1.00
Family migraine history (*n*, %)	8, 21.6%	9, 20.9%	χ^2^: 0.0; *p* = 1.00
Psychological disorder (*n*, %)	8, 21.6%	2, 4.7%	**χ^2^: 3.8; *p*** = **0.05**
Family psychological disorder (*n*, %)	7, 18.9%	4, 9.3%	χ^2^: 0.6; *p* = 0.43
Number of prior concussions (Md [IQR])	1.0 [0.0–1.0]	0.0 [0.0–1.0]	**U: 596.0; *p*** = **0.05**
**Injury characteristics**			
Injury setting (*n*, %)			χ^2^: 0.7; *p* = 0.42
Game	21, 56.8%	21, 48.8%	
Practice	10, 27.0%	5, 11.6%	
Unknown	6, 16.2%	17, 39.5%	*∗NA*
Mechanism of injury (*n*, %)			**χ^2^: 9.2; *p*** = **0.03**
Player to surface	13, 35.1%	12, 27.9%	
Player to player	7, 18.9%	21, 48.8%	
Player to stick	9, 24.3%	5, 11.6%	
Player to Ball/Puck	8, 21.6%	4, 9.3%	
Unknown	0, 0.0%	1, 2.3%	*∗NA*
Loss of consciousness (*n*, %)	5, 13.5%	6, 14.0%	χ^2^: 0.0; *p* = 1.00
Amnesia (*n*, %)	9, 24.3%	3, 7.0%	χ^2^: 3.3; *p* = 0.07
Time to presentation (Md [IQR])	3.0 [2.0–5.0]	2.0 [2.0–5.0]	U: 744.5; *p* = 0.62
Initial PCSS score (Md [IQR])	33.0 [20.0–47.2]	28.0 [8.5–39.5]	U: 380.0; *p* = 0.08
Time to RTL (Md [IQR])	5.0 [2.0–8.2]	3.0 [2.0–5.0]	U: 603.5; *p* = 0.22
Time to SR (Md [IQR])	19.0 [14.0–33.0]	15.0 [5.5–26.0]	U: 343.5; *p* = 0.24
Time to RTP (Md [IQR])	24.0 [19.0–42.5]	20.0 [15.0–50.0]	U: 394.5; *p* = 0.45

Note: ∗NA = not applicable; group excluded from statistical analysis. Md = Median. IQR = Interquartile Range. PCSS = Post-Concussive Symptom Scale. RTL = Return to Learn. SR = Symptom Resolution. RTP = Return to Play. ADHD = Attention-deficit/hyperactivity disorder.

### 3.2 Injury characteristics: setting and contact mechanism

**Fig. 2:**
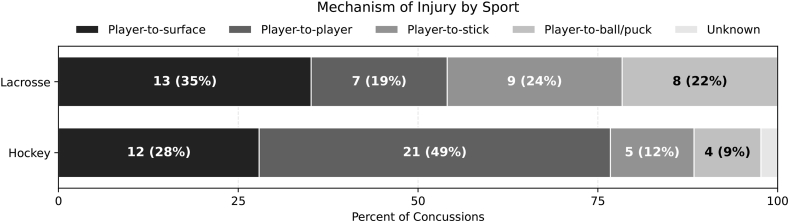
Stacked bar plot of percent of each mechanism of injury per sport.

For lacrosse athletes, 21 (56.8%) of SRCs occurred during a game and 10 (27.0%) occurred during practice, while 6 (16.2%) were unable to be classified. Hockey athletes had a similar distribution of game/practice concussions compared to lacrosse (*p* = *0.42*), with 21 (48.8%) occurring during games, 5 (11.6%) occurring during practice, and 17 (39.5%) unable to be classified. Mechanism of injury differed significantly between the two sports (*p* = *0.03*) (Fig. 2). Compared with lacrosse athletes, hockey athletes were more likely to sustain concussions via player-to-player contact (21/43, 48.8% vs. 7/37, 18.9%) and less likely to have player-to-stick (5/43, 11.6% vs. 9/37, 24.3%) or player-to-ball/puck (4/43, 9.3% vs. 8/37, 21.6%). Player-to-surface injuries were similar in both groups (12/43, 27.9% vs. 13/37, 35.1%). One hockey athlete (2.3%) was unable to be placed in these injury mechanism categories and was excluded from all analyses involving injury mechanism. Little's MCAR test suggests the data that were unable to be classified were missing in a random manner (p = 0.25). The proportion of lacrosse and hockey athletes with loss of consciousness (LOC, *p* = *1.00*) and amnesia (*p* = *0.07*) were similar. Full injury characteristic information can be found in Table 1.

### 3.3 Primary outcome: initial PCSS

**Fig. 3:**
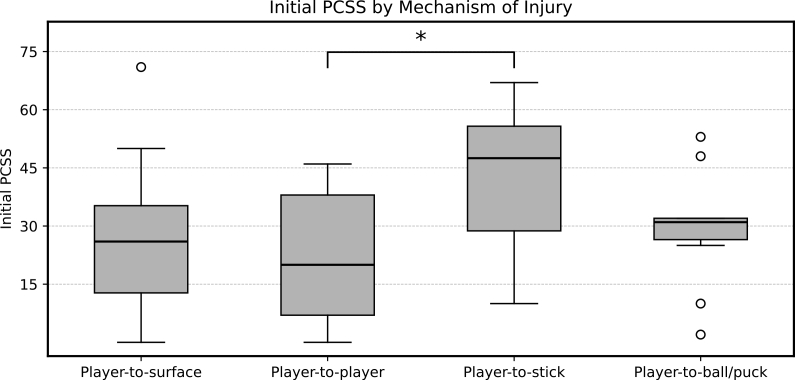
Box plots for initial post-concussion symptom severity score per mechanism of injury.

Time to present to clinic was similar between the lacrosse (median: 3.0 days) and hockey (median: 2.0 days) groups (*p* = *0.62*), indicating that the time to initial PCSS was not significantly different. Lacrosse (median: 33.0) and hockey (median: 28.0) also had similar initial PCSS scores (*p* = *0.08*). Initial PCSS scores were also similar when comparing those injured in a game (median: 33.0) and in practice (median: 29.5; *p* = *0*.*93*) (Table 2). However, there was a significant difference when comparing between those with player-to-surface (median: 26.0), player-to-player (median: 20.0), player-to-stick (median: 47.5), and player-to-ball/puck (median: 31.0) mechanisms of injury (*p* = *0.02*) (Table 2, Fig. 3). Post-hoc analysis indicated that player-to-stick concussions had greater symptom severity compared to player-to-player injuries (*p* = *0.02*), and player-to-stick concussions trended toward having a greater symptom burden compared to player-to-surface injuries (*p* = *0.06*). A multivariable regression model predicting initial PCSS score was significant [F (7, 49) = 5.9, *p* < *0.01*] and explained 38% of the variance in initial PCSS (adj R^2^: 0.38). Older age at concussion (+2.4 per year; 95%CI: 0.2–4.5, *p* = *0.03*) and more prior concussions (+8.3 per concussion, 95%CI: 3.8–12.8, *p* < *0.01*) were independent predictors of a higher initial PCSS. Sport and injury mechanism were not significant predictors. Complete regression data is in Table 3.

**Table 2:** Bivariate analysis of initial symptom severity and recovery timelines based on injury setting and mechanism of injury

	Initial PCSS (Md [IQR])	n	Time to RTL (Md [IQR])	*n*	Time to SR (Md [IQR])	*n*	Time to RTP (Md [IQR])	*n*
Injury setting								
Game	33.0 [19.8–44.2]	36	3.0 [2.0–8.2]	40	17.0 [14.0–33.0]	29	24.0 [19.0–55.0]	29
Practice	29.5 [14.0–48.0]	10	5.0 [1.5–7.0]	15	20.0 [12.0–42.0]	13	22.0 [17.5–42.5]	14
U value; p	U: 176.0; *p* = 0.93		U: 280.0; *p* = 0.71		U: 180.5; *p* = 0.84		U: 166.5; *p* = 0.35	
Mechanism of injury								
Player to surface	**26.0 [12.8**–**35.2]**	20	2.0 [2.0–3.0]	22	12.0 [4.0–16.0]	15	19.0 [7.8–25.5]	16
Player to player	**20.0 [7.0**–**38.0]**^a^	19	4.0 [2.0–7.5]	27	15.0 [7.0–23.8]	19	19.0 [15.5–29.0]	19
Player to stick	**47.5 [28.8**–**55.8]**^a^	14	6.0 [3.2–9.8]	14	20.0 [16.0–33.0]	13	29.0 [22.0–55.0]	13
Player to Ball/Puck	**31.0 [26.5**–**32.0]**	10	4.5 [1.0–8.2]	12	20.0 [13.5–37.5]	11	24.0 [17.5–48.5]	11
H-value; p	**H: 9.6; *p*** = **0.02**		H: 6.6; *p* = 0.09		H: 6.5; *p* = 0.09		H: 5.4; *p* = 0.14	

Md = Median. IQR = Interquartile Range. PCSS = Post-Concussive Symptom Scale. RTL = Return to Learn. SR = Symptom Resolution. RTP = Return to Play.

a Significant post-hoc Dunn Test with Holm Correction, *p* = 0.02.

**Table 3:** Multivariable regression analysis of factors predictive of initial post-concussion symptom severity score, and time to return-to-learn, symptom resolution, and return-to-play

	Β (95% CI)	SE	*p*
**Initial PCSS: F(7, 49)** = **5.9, *p***<**0.01; adj R^2^: 0.38**			
**Age at concussion**	**2.4 (0.2–4.5)**	**1.1**	**0.03**
**Number prior concussions**	**8.3 (3.8–12.8)**	**2.2**	<**0.01**
Female sex (Ref: Male)	6.6 (−3.2–16.5)	4.9	0.18
Lacrosse (Ref: Hockey)	3.5 (−5.1–12.0)	4.3	0.42
Player to player (Ref: Player to surface)	0.7 (−10.1–11.4)	5.4	0.90
Player to stick (Ref: Player to surface)	7.4 (−4.2–19.0)	5.8	0.21
Player to Ball/Puck (Ref: Player to surface)	−0.1 (−14.3–14.0)	7.0	0.99
Time to RTL: F (7, 61) = 1.8, *p* = 0.10; adj R^2^: 0.08			
Age at concussion	0.3 (−0.7–1.3)	0.5	0.52
Number prior concussions	1.4 (−0.4–3.3)	0.9	0.13
Female sex (reference: Male)	−2.4 (−6.9–2.0)	2.2	0.28
Lacrosse (Ref: Hockey)	2.3 (−1.4–5.9)	1.8	0.23
Player to player (Ref: Player to surface)	3.7 (−0.6–7.9)	2.1	0.09
Player to stick (Ref: Player to surface)	3.0 (−2.1–8.0)	2.5	0.24
**Player to Ball/Puck (Ref: Player to surface)**	**5.9 (0.4–11.4)**	**2.7**	**0.03**
Time to SR: F (7, 45) = 0.7, *p* = 0.64; adj R^2^: −0.04			
Age at concussion	−1.3 (−5.9–3.2)	2.2	0.55
Number prior concussions	7.9 (−0.6–16.4)	4.2	0.07
Female sex (Ref: Male)	3.3 (−15.5–22.2)	9.4	0.73
Lacrosse (Ref: Hockey)	−1.3 (−17.9–15.3)	8.2	0.87
Player to player (Ref: Player to surface)	−2.4 (−23.8–19.0)	10.6	0.82
Player to stick (Ref: Player to surface)	3.5 (−20.4–27.4)	11.9	0.77
Player to Ball/Puck (Ref: Player to surface)	2.9 (−21.5–27.2)	12.1	0.81
Time to RTP: F (7, 47) = 1.3, *p* = 0.29; adj R^2^: 0.03			
Age at concussion	−1.6 (−5.8–2.7)	2.1	0.47
**Number prior concussions**	**10.4 (2.1–18.6)**	**4.1**	**0.02**
Female sex (Ref: Male)	4.8 (−13.5–23.2)	9.1	0.60
Lacrosse (Ref: Hockey)	−4.0 (−20.2–12.1)	8.0	0.62
Player to player (Ref: Player to surface)	−8.0 (−28.1–12.0)	10.0	0.43
Player to stick (Ref: Player to surface)	2.0 (−21.2–25.3)	11.6	0.86
Player to Ball/Puck (Ref: Player to surface)	−2.3 (−25.8–21.3)	11.7	0.85

PCSS = Post-Concussive Symptom Scale. RTL = Return to Learn. SR = Symptom Resolution. RTP = Return to Play. Ref = Reference.

### 3.4 Secondary outcome: recovery timeline

The time from injury for an athlete to RTL was similar between lacrosse (median: 5.0) and hockey (median: 3.0; *p* = *0.22*) (Table 1) and between games (median: 3.0) and practices (median: 5.0; *p* = *0.71*) (Table 2). There was a trend for longer time to RTL in those with stick injuries (median: 6.0) compared to those with surface contact (median: 2.0), player contact (median: 4.0), and ball/puck (median 4.5; *p* = *0.09,* Table 2). The time to SR was similar between lacrosse (median: 19.0) and hockey (median: 15.0, *p* = *0.24*, Table 1). Time to SR was also similar between games (median: 17.0) and practice (median: 20.0, *p* = *0.84*, Table 2). There was a trend for longer time to SR in those with stick injuries (median: 20.0) and ball/puck injuries (median: 20.0) than surface contact (median: 12.0) and player contact (median: 15.0; *p* = *0.09*, Table 2). Time to RTP was similar between lacrosse (median: 24.0) and hockey (median: 20.0, *p* = *0.45*, Table 1) and between games (median: 24.0) and practice (median: 22.0, *p* = *0.35*, Table 2). Time to RTP was not significantly different across injury mechanisms (*p* = *0.14*, Table 2). Multivariable regression models to predict time to RTL, time to SR, and time to RTP were not significant (Table 3), though ball/puck injuries predicted an extra week until RTL (+5.9 days, 95% CI: 0.4–11.4, *p* = *0.03*) relative to surface contact. Given that the overall RTL regression model was not statistically significant, this isolated association may reflect a real finding, chance result, or model instability.

### 3.5 Sex based sub-analyses

Among male athletes, lacrosse and hockey players demonstrated similar distributions of game vs practice injuries, mechanisms of injury, initial PCSS scores, and recovery timelines (all *p* > *0.05*). The most common injury mechanism in female athletes was player-to-stick (33.3%), followed by player-to-surface (28.6%) and player-to-ball/puck (23.8%). Player-to-player contact was rare among female athletes (9.5%). Complete data are presented in Table 4.

**Table 4:** Sex-based sub-analysis of injury characteristics, presenting severity, and recovery timeline in lacrosse and hockey players

	Male Lacrosse (*n* = 22)	Male Hockey (*n* = 37)	Statistics
Injury setting (*n*, %)			χ^2^: 0.0; *p* = 1.00
Game	15, 68.2%	20, 54.1%	
Practice	3, 13.6%	5, 13.5%	
Unknown	4, 18.2%	12, 32.4%	*∗NA*
Mechanism of injury (*n*, %)			χ^2^: 3.6; *p* = 0.31
Player to surface	7, 31.8%	12, 32.4%	
Player to player	7, 31.8%	19, 51.4%	
Player to stick	4, 18.2%	3, 8.1%	
Player to Ball/Puck	4, 18.2%	3, 8.1%	
Initial PCSS (Md [IQR])	25.0 [18.0–34.0]	20.0 [4.0–37.0]	U: 211.5; *p* = 0.42
Time to RTL (Md [IQR])	3.0 [2.0–10.0]	3.0 [2.0–5.0]	U: 302.5; *p* = 0.27
Time to SR (Md [IQR])	25.0 [14.0–37.5]	12.0 [5.0–19.0]	U: 132.0; *p* = 0.12
Time to RTP (Md [IQR])	30.0 [19.0–42.5]	20.0 [15.0–28.5]	U: 159.5; *p* = 0.26

	**Female lacrosse (*n*** = **15)**	**Female hockey (*n*** = **6)**	**Statistics**

Injury setting (*n*, %)			*∗NA*
Game	6, 40.0%	1, 16.7%	
Practice	7, 46.7%	0, 0.0%	
Unknown	2, 13.3%	5, 83.3%	
Mechanism of injury (*n*, %)			*∗NA*
Player to surface	6, 40.0%	0, 0.0%	
Player to player	0, 0.0%	2, 33.3%	
Player to stick	5, 33.3%	2, 33.3%	
Player to Ball/Puck	4, 26.7%	1, 16.7%	
Unknown	0, 0.0%	1, 16.7%	
Initial PCSS (Md [IQR])	46.0 [34.0–49.0]	35.0 [27.2–40.5]	*∗NA*
Time to RTL (Md [IQR])	5.0 [2.5–7.0]	5.0 [3.0–6.0]	*∗NA*
Time to SR (Md [IQR])	17.0 [13.0–21.0]	35.0 [11.2–79.0]	*∗NA*
Time to RTP (Md [IQR])	21.0 [18.0–35.5]	40.0 [17.0–80.2]	*∗NA*

Note. ∗NA = not applicable; group was excluded from statistical analysis.

Md = Median. IQR = Interquartile Range. PCSS = Post-Concussive Symptom Scale. RTL = Return to Learn. SR = Symptom Resolution. RTP = Return to Play.

## 4 Discussion

### 4.1 Summary of findings

This retrospective cohort study examined whether sport, injury mechanisms, or competition setting impacted initial symptom burden and recovery trajectories between youth and adolescent lacrosse and hockey athletes with sport-related concussions. A greater proportion of the hockey sample was male, while a greater proportion of the lacrosse sample had a pre-injury psychiatric history and a higher number of prior concussions. When comparing both stick-based sports to each other, both hockey and lacrosse athletes had comparable initial symptom severities and similar times to RTL, SR, and RTP. However, hockey players were more likely to be injured in player-to-player contact, whereas lacrosse athletes were more evenly divided among contact mechanisms. Across the combined cohort, head-to-stick-related concussions had the highest initial symptom burden, indicating a potentially more severe mechanism. Additionally, ball or puck impacts may be associated with longer time to return to learn compared with surface contact; however, because the overall RTL model was not statistically significant and only this single covariate reached nominal significance, this finding may be unstable or spurious and should be interpreted cautiously. While sport and injury mechanism were not predictive of initial symptom scores in multivariable regression, older age and greater number of prior concussions were predictive of higher initial symptom scores. In a male-only sub-analysis, lacrosse and hockey athletes again showed similar mechanisms, symptom burden, and recovery timelines.

### 4.2 Impact of injury mechanism

In our cohort, concussions in lacrosse arose from a more balanced mix of equipment- and contact-related mechanisms, whereas hockey concussions were driven primarily by player-to-player contact, consistent with prior reports in stick-handling sports. These mechanism patterns likely reflect intrinsic differences in playing environment and contact rules between the sports. Ice hockey occurs on a low-friction surface with skates, resulting in increased player speed and momentum. Play is also confined in a rink, where athletes may be funneled toward opponents and the boards. These conditions may amplify opportunities for high-energy player-to-player contact and player-to-surface impacts [19]. In contrast, lacrosse is played on an open field where collisions may be more spatially dispersed. As the ball more regularly moves through the air, there is increased opportunity for head impacts from equipment. Together these sport-specific features may explain the differences in injury mechanism frequency.

Prior literature supports this pattern. For example, Wilcox et al. found that most head impacts in collegiate hockey players occurred due to player-to-player contact [19]. In high school boy's lacrosse, Lincoln et al. found that player-to-player was also the most common cause of concussion [15]. However, in high school girls' lacrosse athletes, Caswell et al. found that stick injuries were the most common cause of concussions [10]. In larger epidemiologic studies, these mechanism rates are consistent across all injuries: male hockey and lacrosse athletes are more often injured by player contact, while female athletes are more often injured by the stick or ball [5,8]. This likely reflects differences in rules, officiating, and sport culture: physical contact is more integral to boys' lacrosse and ice hockey, while girls' lacrosse limits intentional body contact [21,22]. When limiting analysis to male athletes only, injury mechanism between the two sports was similar and predominantly player-to-player or player-to-surface based, similar to existing literature [5,8,15,19]. These findings together suggest that sex-specific contact rules and sport culture largely determine whether concussions arise from player contact or equipment.

Injury mechanism was also found to influence initial symptom burden. Stick-related injuries and, to a lesser extent, ball/puck injuries were associated with higher symptom scores. While few studies directly link mechanism to acute symptom burden in stick-handling sports, these findings are consistent with biomechanical work. One plausible explanation may relate to the biomechanical principle of impulse, which is defined as the force applied over the time [23]. Sticks and balls/pucks can reach high velocities and strike a relatively small surface area, often unexpectedly, resulting in a high-magnitude, focal impact delivered over a short contact duration [[24], [25], [26]]. In contrast, player-to-player collisions tend to distribute force across a higher body surface and longer contact duration. Differences in the force–time profile may explain why equipment-related mechanisms were associated with greater initial symptom burden in our cohort. Future work may further explore how the biomechanical forces generated by equipment compare to distributed player-to-player contact in youth sports.

### 4.3 Clinical implications

In addition to higher initial symptom burden, equipment-based injuries—from a stick or ball/puck—also trended towards longer time to return-to-learn and symptom resolution compared to surface or player contact. Projectile injuries (i.e., ball/puck) were associated with an additional week recovery before athletes returned to learn when compared to surface-based injuries; however, due to the overall regression model lacking statistical significance, this association should be interpreted cautiously. Prior literature has also shown higher acute symptoms burden is one of the strongest predictors of a delayed return to school, which tends to occur when symptoms begin to subside [27]. In the context of stick-handling sports, where concussions can often arise from the stick or ball/puck, equipment-based concussions may warrant closer earlier monitoring and additional anticipatory guidance. Identifying this subgroup earlier may facilitate the allocation of additional resources and school accommodations for athletes more likely to miss additional days of learning.

### 4.4 Limitations

This was a retrospective analysis from a single specialty sports concussion clinic, which may introduce selection bias. Athletes who present to a specialty concussion clinic are more likely to represent a subset of all concussed athletes, such as those with a potentially more severe or complex injury [28], greater access to care [29], or differences in care-seeking behavior including greater parental involvement, prior concussion history, or more psychological comorbidity [30,31]. In addition, these athletes may have been initially seen by local providers and somehow mismanaged, making their recovery timeline atypical. As such, these findings may not be generalizable to all athletes with sport-related concussions, particularly those managed outside of specialty care settings. Furthermore, the overall sample and subgroup sizes were modest, limiting power and increasing the risk of type II error. Multivariable models included 7 predictors, yielding as few as 7–8 observations per predictor in some models which can increase the risk of overfitting or unstable coefficient estimates. The precision of mechanism- and sex-specific estimates may be limited. While the inclusion of these covariates (age, sex, prior concussion history) was based on their documented influence on concussion symptom severity and recovery [32,33], these results should be considered hypothesis-generating rather than confirmatory. In addition, mechanism and setting were extracted from clinical documentation, which relied on both patient recall of the injury and provider-recorded descriptions at the time of visit. Not all patients had definitive documentation, resulting in missing data. Therefore, complete-case regression further reduced sample size, which may have introduced bias if missing data correlated with other variables (i.e. sex, sport, etc.). That said, several prior papers have relied on this suboptimal methodology [28,34,35], which highlights our desire to follow the best available and evidence-based practices. Although Little's MCAR test supported that data were missing in a random pattern, this assumption should still be interpreted with caution. Additionally, lacrosse and hockey differed on sex, psychiatric history, and prior concussion history. While sport was modelled in our statistical analyses, there is a possibility that some results may be influenced by sport-specific factors. Finally, outcomes were based on self-reported symptom scales and clinician-documented milestones rather than standardized testing for all athletes. Larger, prospective, multicenter studies with standardized metrics will help confirm these patterns and more precisely define how sport, mechanism, and individual factors interact to shape concussion recovery in stick-handling sports.

### 4.5 Conclusion

In a cohort of lacrosse and hockey players with sport-related concussion, recovery timelines and symptom severity were similar. However, equipment-based injury mechanisms resulted in higher initial symptom burden. Therefore, stick and ball/puck related concussions may warrant more initial support, while overall prognosis is driven holistically by individual risk factors, including age, sex, and prior concussion history.

## Declaration of generative AI and AI-assisted technologies in the manuscript preparation process

During the preparation of this work the author(s) used OpenAI's ChatGPT in order to structure statistical analysis and support grammar. Google's Gemini was also used to create Fig. 1. After using this tool/service, the author(s) reviewed and edited the content as needed and take(s) full responsibility for the content of the published article.

## Funding

None.

## Declaration of competing interest

The authors declare the following financial interests/personal relationships which may be considered as potential competing interests:

• Douglas Terry, Ph.D., serves as a consultant/Senior Director of Research for the National Football League (NFL) and a scientific advisor for HitIQ. He previously consulted for REACT Neuro, Inc. He has a consulting practice in forensic neuropsychology, including expert testimony, involving individuals who have sustained mild TBIs (including former athletes). He receives grant funding from Amgen, Inc. and Football Research, Inc.

• Scott Zuckerman, MD, MPH, is a member of the NFL Head, Neck, and Spine Committee and a consultant for Medtronic.

• No other authors have any declarations or conflicts of interest to report.

## References

[1] Lincoln A.E., Hinton R.Y., Almquist J.L. (2007). Head, face, and eye injuries in scholastic and collegiate lacrosse: a 4-year prospective study. Am J Sports Med.

[2] Marar M., McIlvain N.M., Fields S.K. (2012). Epidemiology of concussions among United States high school athletes in 20 sports. Am J Sports Med.

[3] Halstead M.E., Walter K.D., Moffatt K. (2018). Sport-Related concussion in children and adolescents. Pediatrics.

[4] Kerr Z.Y., Lincoln A.E., Dodge T. (2018). Epidemiology of Youth Boys' and Girls' lacrosse Injuries in the 2015 to 2016 seasons. Med Sci Sports Exerc.

[5] Yard E.E., Comstock R.D. (2006). Injuries sustained by pediatric ice hockey, lacrosse, and field hockey athletes presenting to United States emergency departments, 1990-2003. J Athl Train.

[6] Potvin B.M., Aguiar O.M.G., Komisar V. (2019). A comparison of the magnitude and duration of linear and rotational head accelerations generated during hand-, elbow- and shoulder-to-head checks delivered by hockey players. J Biomech.

[7] West S.W., Pankow M.P., Gibson E.S. (2023). Injuries in Canadian high school boys' collision sports: insights across football, ice hockey, lacrosse, and rugby. Sport Sci Health.

[8] Xiang J., Collins C.L., Liu D. (2014). Lacrosse injuries among high school boys and girls in the United States: academic years 2008-2009 through 2011-2012. Am J Sports Med.

[9] Bowers A.L., Horneff J.G., Baldwin K.D. (2010). Thumb injuries in intercollegiate men's lacrosse. Am J Sports Med.

[10] Caswell S.V., Lincoln A.E., Almquist J.L. (2012). Video incident analysis of head injuries in high school girls' lacrosse. Am J Sports Med.

[11] Zetterberg H., Winblad B., Bernick C. (2019). Head trauma in sports - clinical characteristics, epidemiology and biomarkers. J Intern Med.

[12] Hallock H., Mantwill M., Vajkoczy P. (2023). Sport-Related concussion: a cognitive perspective. Neurol, Clin Pract.

[13] Black A.M., Meeuwisse D.W., Eliason P.H. (2021). Sport participation and injury rates in high school students: a Canadian survey of 2029 adolescents. J Saf Res.

[14] Kerr Z.Y., Roos K.G., Lincoln A.E. (2019). Injury incidence in youth, high school, and NCAA men's lacrosse. Pediatrics.

[15] Lincoln A.E., Caswell S.V., Almquist J.L. (2013). Video incident analysis of concussions in boys' high school lacrosse. Am J Sports Med.

[16] Hinton R.Y., Lincoln A.E., Almquist J.L. (2005). Epidemiology of lacrosse injuries in high school-aged girls and boys: a 3-year prospective study. Am J Sports Med.

[17] Ling D.I., Cheng J., Santiago K. (2020). Women are at higher risk for concussions due to ball or equipment contact in soccer and lacrosse. Clin Orthop Relat Res.

[18] Abed V., Hawk G.S., Akarakian R. (2023). Epidemiological analysis of concussions in youth ice hockey players: a national emergency department database study. Am J Emerg Med.

[19] Wilcox B.J., Machan J.T., Beckwith J.G. (2014). Head-impact mechanisms in men's and women's collegiate ice hockey. J Athl Train.

[20] Google. Gemini. https://gemini.google.com/app.

[21] Houghton K.M., Emery C.A., Canadian Paediatric S. (2012). Bodychecking in youth ice hockey. Paediatr Child Health.

[22] Barber Foss KD., Le Cara E., McCambridge T. (2018). Epidemiology of injuries in women's lacrosse: implications for Sport-, Level-, and sex-specific injury prevention strategies. Clin J Sport Med.

[23] Meaney D.F., Smith D.H. (2011). Biomechanics of concussion. Clin Sports Med.

[24] Post A., Dawson L., Hoshizaki T.B. (2019). The influence of impact source on variables associated with strain for impacts in ice hockey. Comput Methods Biomech Biomed Eng.

[25] Post A., Hoshizaki T.B., Karton C. (2019). The biomechanics of concussion for ice hockey head impact events. Comput Methods Biomech Biomed Eng.

[26] Post A., Hoshizaki T.B., Gilchrist M.D. (2017). Peak linear and rotational acceleration magnitude and duration effects on maximum principal strain in the corpus callosum for sport impacts. J Biomech.

[27] Iverson G.L., Terry D.P., Maxwell B. (2022). Greater acute concussion symptoms are associated with longer recovery times in NCAA division III collegiate athletes. Front Neurol.

[28] Hou B.Q., Yengo-Kahn A.M., Hajdu K. (2022). Factors associated with additional Clinic visits in the treatment of sports-related concussion. Clin J Sport Med.

[29] Corwin D.J., Fedonni D., McDonald C.C. (2024). Community and patient features and health care point of entry for pediatric concussion. JAMA Netw Open.

[30] Warmath D., Winterstein A.P., Myrden S. (2022). Parents and coaches as transformational leaders: motivating high school athletes' intentions to report concussion symptoms across socioeconomic statuses. Soc Sci Med.

[31] van Ierssel J.J., Kutcher S.A., Johnston S. (2026). Prevalence and risk of anxiety and depression after concussion: a TRANSCENDENT Study. J Neurotrauma.

[32] Levin H.S., Temkin N.R., Barber J. (2021). Association of sex and Age with mild traumatic brain injury–related symptoms: a TRACK-TBI Study. JAMA Netw Open.

[33] Cuff S., Maki A., Feiss R. (2022). Risk factors for prolonged recovery from concussion in young patients. Br J Sports Med.

[34] Bishay A.E., Albert A.N., Rigney G.H. (2025). Does mechanism of injury affect recovery after sport-related concussion in basketball? A pilot Study. Neurosurgery.

[35] De Oliveira N., Horsey J., Vanleuven J. (2025). Does the presence of acute sleep initiation symptoms impact recovery from sport-related concussion?. Brain Inj.

